# Cancer immunology and immunotherapy

**DOI:** 10.20892/j.issn.2095-3941.2021.0036

**Published:** 2021-11-24

**Authors:** Xiubao Ren

**Affiliations:** 1 Department of Immunology, Tianjin Medical University Cancer Institute and Hospital, National Clinical Research Center for Cancer, Tianjin 300060, China; 2 Department of Biotherapy, Tianjin Medical University Cancer Institute and Hospital, National Clinical Research Center for Cancer, Tianjin 300060, China; 3 Key Laboratory of Cancer Prevention and Therapy, Tianjin’s Clinical Research Center for Cancer, Key Laboratory of Cancer Immunology and Biotherapy, Tianjin 300060, China

At the beginning of the last century, Dr. William Coley introduced “Coley’s toxin” to the scientific community after observing that a small number of patients with sarcoma with infections developed spontaneous tumor regression. “Coley’s toxin” consisted of bacteria or bacterial products and achieved a durable response in a small group of patients with cancer, most of whom had inoperable sarcoma. However, this response was usually unpredictable because of the lack of understanding of the immune response at that time. Since then, researchers have continued to study the relationship between cancer and the host immune system, and the discovery of “Coley’s toxin” marked the beginning of cancer immunotherapy^1^.

More than half a century after Coley’s initial discovery, Steven A. Rosenberg introduced interleukin (IL)-2 and tumor-infiltrating lymphocytes to the field of cancer immunology and immunotherapy. Tumor-infiltrating lymphocytes, lymphokine-activated killer cells, and cytokine-induced killer (CIK) cells were the cornerstones of this new pillar of cancer immunotherapy^1^.

The breakthrough in cancer immunotherapy came after the discovery of immune checkpoints—negative regulators of the immune response—and the development of gene modification technologies, which eventually led to the development of immune checkpoint inhibitors (ICIs) and genetically modified immune cells, such as T-cell receptor-engineered T-cells (TCR-T) and chimeric antigen receptor T cells (CAR-T), which have since achieved clinical success in a variety of cancers.

We are currently living in an era of cancer immunotherapy. Several ICIs and CAR-T cell therapies have already been approved by regulatory agencies for the treatment of cancer. Although the applications of these therapies are limited mainly to patients with refractory or relapsed cancer, many clinical trials have already begun to examine the roles of immunotherapy as first-line treatments.

However, despite encouraging success of immunotherapies in clinical trials, a substantial proportion of cancer patients still do not show durable responses to treatment. In addition, immune-related adverse events (irAEs) have emerged as a critical issue that must be considered in clinical practice. Therefore, identification of biomarkers to predict clinical responses and irAEs for immunotherapies is urgently required^2^.

In this column, which we are honored to host, Yin et al. and Gong et al. suggest novel candidates for biomarkers that predict the clinical benefit of ICIs. These biomarkers include the gut microbiome and mutations in DNA damage response or mismatch repair genes, both of which are current research hotspots. In contrast, Bai et al., also featured in this column, analyze the associations among irAEs and clinical baseline characteristics, routine clinical examination indicators, and peripheral immune cell subgroups during ICIs treatment. In addition, Zhang et al. highlight ferritin as a distinct diagnostic and prognostic biomarker for irAEs. Increasing evidence indicates that tertiary lymphoid structure (TLS) in tumor tissue is a key determinant of response to ICIs^3^. Also in this column, Zhao et al. summarize recent research advances in TLS in human solid tumors, particularly the associated antitumor mechanisms and TLS-based therapeutic approaches.

Combination therapy with other medications is an effective way to further improve the efficacy of ICIs. In this column, Tang et al. and Ye et al. describe various strategies for applying ICIs in combination therapy. Even some controversial drugs in the treatment of cancer may also have a role in the combination regimen using ICIs. For instance, a thought-provoking editorial by Yingyan Yu enlightens us that glucocorticoids can also be applied as adjuvant reagents for ICIs in solid cancers. These findings deepen the understanding of ICIs, facilitate appropriate selection, and maximize clinical benefits for cancer patients.

Cell therapy, such as CAR-T therapy, has also achieved success in clinical trials in treating several types of malignancies, particularly hematological malignancies. Xu et al. provide a review of cellular immunotherapy for hematological malignancy in this column. Unlike ICIs, CAR-T, normally involving genetic modification of the patient’s own immune cells, is not yet an off-the-shelf agent. Its cost may limit patient access to this novel treatment. Furthermore, not all laboratories and clinical centers are qualified to administer CAR-T therapy, owing to the strict requirements of good operating practice procedures and high-end equipment. Han et al. describe a novel method that can generate sufficient CAR-T cells for clinical use from 50 to 100 mL of peripheral blood. Their protocol not only demonstrates that an apheresis machine is not always a requirement for generating CAR-T cells but also suggests that the initial amount of T cells for CAR-T cell preparation may be much lower than previously estimated. This work represents a small but notable step toward overcoming the major hurdles limiting CAR-T therapy in patients.

Among the diverse immune cell therapies, CIK immunotherapy is currently emerging as a promising and effective treatment option. A recent clinical trial has shown that CIK cells significantly improve overall survival and progression-free survival in patients with non-small-cell lung cancer (NSCLC)^4^. Wang et al., the same group that conducted the above clinical trial, have performed whole-exome sequencing on tumor tissues and paired adjacent benign tissues collected from patients with NSCLC as well as RNA-seq on NSCLC tumor tissues before CIK treatment, with the aim of discovering predictive markers for CIK therapy. Their report reveals a distinct landscape of genes and infiltrating immune cells in NSCLC that may aid in understanding of the anticancer mechanisms of CIK immunotherapy.

CIK cells, which usually display effector memory T or terminally differentiated effector memory T phenotypes, exert broad non-major histocompatibility antigen-restricted antitumor activity against a variety of cancer cells, much like natural killer (NK) cells. In contrast to CIK cells, NK cells, members of the innate immune system, are traditionally believed not to have a memory phenotype. Nonetheless, trained immunity of NK cells has recently been proposed. In this column, Zhang et al. identify a unique cluster of NK cells as the precursors of trained NK cells. Furthermore, they report a critical role of the epigenetic regulator EZH2 in the induction of trained immunity in human NK cells.

High tumor mutation burden and mutation-derived neoantigens are significantly associated with improved survival in cancer patients receiving immunotherapy. Ye et al. have developed a protocol to identify and further modify neoepitopes to generate more powerful neoepitope vaccines. Their findings not only shed light on the development of neoantigen-specific tumor vaccines but also are highly instructive for the development of cell therapy targeting neoantigens, such as TCR-T.

Not all immune cells have anti-tumor roles in the tumor microenvironment. CD4^+^CD25^+^ regulatory T cells (Tregs) are major immunosuppressive cells that inhibit CD8^+^ cytotoxic T lymphocytes in the tumor microenvironment. Targeting Tregs is an effective strategy to ease the immunosuppressive tumor microenvironment and thus enhance the efficacy of standard treatments for cancer, as demonstrated by Li et al. Interestingly, recent research has indicated that Foxp3^+^ epithelial cells can mimic the immunosuppressive function of Tregs in the tumor microenvironment. The editorial by Gong et al. provides more details about their notable work. Tumor angiogenesis is another key immunosuppressive factor in tumor microenvironment. Accumulating evidence suggests that vascular normalization might enhance antitumor immunity. An editorial by Wu et al. unveils the role of delta-like 1, a Notch ligand, in long-term vascular normalization and cancer immunotherapy.

As described above, the tumor microenvironment is very complex, comprising various cell types and extracellular matrix. Recently, multiplexing imaging technologies have been developed and emerged as crucial tool to better study the tumor microenvironment. An editorial by Zhang et al. provided in-depth insight into multiplexing imaging technologies and their characteristics, as well as their applications in cancer immune research.

With the rapid progress of knowledge in the life sciences and the availability of novel technologies, such as next-generation sequencing and precision medicine, a new medical philosophy has changed the ways in which cancer care, including immunotherapy, is provided. Kiyotani et al. provide a timely review of personalized immunotherapy in cancer precision medicine in this column.

While we hail a new era of cancer immunotherapy, we must realize that we are still at the very beginning of this therapeutic breakthrough. With immunotherapy now being applied to a broad variety of hematological and solid malignancies, basic research must be further strengthened to decipher the molecular mechanisms underlying the physiological and pathological processes of cancer immunology and to enhance the power of immunotherapy and decrease adverse effects. Cancer immunotherapy is a typical example of successful bench-to-bedside translation, which is, and should always be, based on a solid scientific foundation.

## References

[1] Waldman AD, Fritz JM, Lenardo MJ (2020). A guide to cancer immunotherapy: from T cell basic science to clinical practice. Nat Rev Immunol.

[2] Das S, Johnson DB (2019). Immune-related adverse events and anti-tumor efficacy of immune checkpoint inhibitors. J Immunother Cancer.

[3] Helmink BA, Reddy SM, Gao J, Zhang S, Basar R, Thakur R (2020). B cells and tertiary lymphoid structures promote immunotherapy response. Nature.

[4] Liu L, Gao QL, Jiang JT, Zhang JP, Song X, Cui JW (2020). Randomized, multicenter, open-label trial of autologous cytokine-induced killer cell immunotherapy plus chemotherapy for squamous non-small-cell lung cancer: NCT01631357. Signal Transduct Target Ther.

